# Using Fuzzy Logic to Enhance Stereo Matching in Multiresolution Images

**DOI:** 10.3390/100201093

**Published:** 2010-01-29

**Authors:** Marcos D. Medeiros, Luiz Marcos G. Gonçalves, Alejandro C. Frery

**Affiliations:** 1 DCA-CT-UFRN, Campus Universitário, Lagoa Nova, Universidade Federal do Rio Grande do Norte, 59072-970 Natal RN, Brazil; E-Mail: marcosdumay@dca.ufrn.br; 2 Instituto de Computação, LCCV & CPMAT, Universidade Federal de Alagoas, BR 104 Norte km 97, 57072-970 Maceió AL, Brazil; E-Mail: acfrery@pesquisador.cnpq.br

**Keywords:** image analysis, fuzzy rules, multiresolution, sensor configuration, stereo matching, vision

## Abstract

Stereo matching is an open problem in Computer Vision, for which local features are extracted to identify corresponding points in pairs of images. The results are heavily dependent on the initial steps. We apply image decomposition in multiresolution levels, for reducing the search space, computational time, and errors. We propose a solution to the problem of how deep (coarse) should the stereo measures start, trading between error minimization and time consumption, by starting stereo calculation at varying resolution levels, for each pixel, according to fuzzy decisions. Our heuristic enhances the overall execution time since it only employs deeper resolution levels when strictly necessary. It also reduces errors because it measures similarity between windows with enough details. We also compare our algorithm with a very fast multi-resolution approach, and one based on fuzzy logic. Our algorithm performs faster and/or better than all those approaches, becoming, thus, a good candidate for robotic vision applications. We also discuss the system architecture that efficiently implements our solution.

## 1. Introduction

The goal of stereo vision is to recover 3D information given incomplete and possibly noisy information of the scene [1, 2]. Depth (or shape) is useful for terrain mapping [3], robot controlling [4–7] and several other applications. Shape from shading, structured light and stereoscopy are among the many possible sources of information. In this work we propose enhancements to the determination of matching points in pairs of images, which stems as the bottleneck of the stereo vision process.

Our approach consists of performing an initial coarse matching between low resolution versions of the original images. The result is refined on small areas of increasingly higher resolution, until the matching is done between pixels in the original images resolution level. This is usually termed “coarse to fine” or “cascade correlation”.

Multiresolution procedures can, in principle, be performed in any order, even in a backwards and forwards scheme, but our choice is based upon computational considerations aiming at reducing the required processing time. Multiresolution matching, in particular, is known to reduce the complexity of several classes of image processing applications, including the matching problem, leading to fast implementations. The general problem with multiresolution algorithms is that, more often than not, they start with the coarsest resolution for all pixels and thus spend a long time. Our approach improves the search for an optimal resolution where to find correspondence points.

The main contribution of this work is proposing, implementing and assessing a multiresolution matching algorithm with starting points whose levels depend on local information. Such levels are computed using a new heuristic based on fuzzy decisions, yielding good quality and fast processing.

The paper unfolds as follows. Section 2 presents a review of image matching, focused on the use of multilevel and fuzzy techniques. Section 3 formulates the problem. Section 4 presents the main algorithms, and Section 5 discusses relevant implementation details. Section 6 presents results, and Section 7 closes with the main contributions, drawbacks and possible extensions of this work.

## 2. State of the Art

Vision is so far the most powerful biological sensory system. Since computers appeared, several artificial vision systems have been proposed, inspired by their biological versions, aiming at providing vision to machines. However, the heterogeneity of techniques necessary for modeling complete vision algorithms makes the implementation of a real-time vision system a hard and complex task.

Stereo vision is used to recover the depth of scene objects, given two different images of them. This is a well-defined problem, with several text books and articles in the literature [1, 2, 8–11]. Disparity calculation is the main issue, making it a complex problem. Several algorithms have been proposed in order to enhance precision or to reduce the complexity of the problem [12–16]. Features as depth (or a disparity map) are useful for terrain mapping [3], robot controlling [6, 7, 17] and several other applications.

Stereo matching is generally defined as the problem of discovering points or regions of one image that match points or regions of the other image on a stereo image pair. That is, the goal is finding pairs of points or regions in two images that have local image characteristics most similar to each other [1, 2, 8–10, 18–20]. The result of the matching process is the displacement between the points in the images, or disparity, also called the 2.5D information. Depth reconstruction can be directly calculated from this information, generating a 3D model of the detected objects using triangulation or other mesh representation. Disparity can also be directly used for other purposes as, for instance, real-time navigation [21].

There are several stereo matching algorithms, generally classified into two categories: area matching and/or feature (element) matching [1]. Area matching algorithms are characterized by comparing features distributed over regions. Feature matching uses local features, edges and borders for instance, with which it is possible to perform the matching.

Area based algorithms are usually slower than feature based ones, but they generate full disparity maps and error estimates. Area based algorithms usually employ correlation estimates between image pairs for generating the match. Such estimates are obtained using discrete convolution operations between images templates. The algorithm performance is, thus, very dependent on the correlation and on the search window sizes. Small correlation windows usually generate maps that are more sensitive to noise, but less sensitive to occlusions, better defining the objects [22].

In order to exploit the advantages of both small and big windows, algorithms based on variable window size were proposed [3, 22, 23]. These algorithms trade better quality of matching for shorter execution time. In fact, the use of full resolution images fairly complicates the stereo matching process, mainly if real time is a requirement.

Several models have been proposed in the literature for image data reduction. Most of them treat visual data as a classical pyramidal structure. The scale space theory is formalized by Witkin [24] and by Lindeberg [25]. The Laplacian pyramid is formally introduced by Burt and Adelson [26], but its first use in visual search tasks is by Uhr [27]. Several works use it as input, mainly for techniques that employ visual attention [28, 29].

Wavelets [30] are also used for building multiresolution images [31], with applications in stereo matching [32–34]. Other multiresolution algorithms have also been used for the development of real-time stereo vision systems, using small (reduced) versions of the images [35, 36].

Multiresolution algorithms mix both area and feature matching for achieving fast execution [34, 37]. Multiresolution matching can even reduce the asymptotic complexity of the matching problem, but at the expense of worse results.

Besides the existence of these *direct* algorithms, Udupa [38] suggests that approaches based on fuzzy sets should be taken into consideration, considering the fact that images are inherently fuzzy. Such approach should be able to handle realistically uncertainties and heterogeneity of object properties.

Several works use logic fuzzy clustering algorithms in stereo matching in order to accelerate the correspondence process [39–46]; some of these technique achieve real time processing. The idea is to pre-process images, group features by some fuzzy criteria or guide the search so the best match between features can be determined, or at least guided, using a small set of candidate features. Fuzzy logic for object identification and feature recovering on stereo images and video is also used [47–50].

Fuzzy theory is also applied to determine the best window size with which to process correlation measures in images [51]. This is in certain degree related to our work, since we determine the best resolution level to start stereo matching, which means determining window size if only one level of resolution would be used. Fuzzy techniques have also been used in tracking and robot control with stereo images [52–54].

Our proposed approach is rather different from the above-listed works and integrates multiresolution procedures with fuzzy techniques. As stated above, the main problem with the multiresolution approach is how to determine the level with which to start correlation measures. A second problem is that, even if a good level is determined for a given pixel, this will not be the best for all the other image pixels, because this issue is heavily dependent on local image characteristics. So, we propose the use of fuzzy rules in order to determine the optimal level for each region in the image. This proposal leads to the precise determination of matching points in real time, since most of the image area is not considered in full resolution.

Our algorithm performs faster and better than plain correlation, and it presents improved results with respect to a very fast multi-resolution approach [17], and one based on fuzzy logic [41].

This paper extends results by Medeiros and Gonçalves [55] by presenting an updated literature review, by a more detailed discussion and explanation about the proposed technique and by the presentation and discussion of further results.

## 3. Stereo Matching Problem

In the stereo matching problem, we have a pair of pictures of the same scene taken from different positions, and possibly orientations, and the goal is to discover corresponding points, that is, pixels in both images that are projections of the same scene point. The most intuitive way of doing that is by comparing groups of pixels of the two images to obtain a similarity value. After similarities are computed, one may or may not include restrictions and calculate the matching that maximizes the global similarity. Our proposal assumes (i) continuity of disparity, and (ii) uniqueness of the correct matching.

In general, given a point in one image, the comparison is not made with all points of the other image. Using the epipolar restriction [2, 16], only pixels on a certain line in one image are the corresponding candidates of a pixel in the other one. The orientation of this line depends only of the relative orientation of the two cameras. The test images used in the current work have a horizontal epipolar line, thus pixels are searched only in such direction.

We measure similarity with the normalized sample cross correlation between images *x* = (*x*(*i*, *j*))_1≤*i*≤*m*,1≤*j*≤*n*_ and *y* = (*y*(*i*, *j*))_1≤*i*≤*m*,1≤*j*≤*n*_, estimated by the linear Pearson correlation coefficient as

(1) $$r_{x,y}=\frac{n\sum_{i,j}\;[x(i,j)y(i,j)]-[\sum_{i,j}\;x(i,j)][\sum_{i,j}y(i,j)]}{\sqrt{n\sum_{i,j}\;{\left[x(i,j)\right]}^{2}-{\left[\sum_{i,j}\;x(i,j)\right]}^{2}}\sqrt{n\sum_{i,j}\;{\left[y(i,j)\right]}^{2}-{\left[\sum_{i,j}y(i,j)\right]}^{2}}}.$$

If the objects are known to lie within a distance range, the search for the best match can be restricted to a subset of the epipolar line. We will refer to this subset as the “search interval”, to avoid confusion with the refining interval that will be defined latter.

Small search intervals, if can be defined, improve the quality of the resulting matching and avoid false positives that are far from the desired match on the epipolar line. While for many problems this is convenient, for some, remarkably in robotic vision, near objects are the most important ones, requiring thus a full matching between the images.

### 3.1. Plain correlation algorithm

We compare here the plain correlation and multiresolution matching approaches. Both algorithms have as common attribute the window size. Although some authors recommend the use of a 7 × 7 window for plain correlation (see, for instance, the work of Hirshmuller [22]), we opted for testing several window sizes in order to compare the relative performances of both approaches.

Traditional plain correlation calculates the normalized, linear cross correlation between all possible windows of both images. For each point in one image, the matching point is chosen in the other image such as to maximize the correlation coefficient.

When matching square images of side *w*, this algorithm calculates *w*^3^ correlations, but when a search interval *w_s_ < w* is available, the number of correlations drops down to *w_s_w*^2^. Of course, in the worst case, we should assume that the plain correlation approach would have *O*(*w*^3^) complexity.

### 3.2. Multiresolution matching with fixed depth

Multi-resolution stereo matching uses several pairs of images of the same scene, sampled with different levels of detail, as a double pyramidal representation of the scene [17]. As in any scale space, images at the base of the pyramid have higher resolution and, therefore, more detail of the scene than those at the top. The credit for using this idea in visual tasks can be given to Uhr [27]. The scale space theory is formalized by Witkin [24], and further by Lindeberg [25]. A variation, the Laplacian pyramid, was introduced by Burt and Adelson [26]. Tsotsos [56, 57] integrated multi-resolution into visual attention, implemented as such by Burt [58], and used in several visual models [28, 29, 59, 60]. Based on multi-resolution, Lindeberg [61] detected features using an automatic scale selection algorithm, while Lowe [62] dealt with detection of scale-invariant features.

Multiresolution algorithms in stereo matching calculate the disparity of all pixels (or blocks of pixels) of a coarse level image and refine them, matching the pixels of finer level images with a small number of pixels around the coarser match. We refer to the interval that contains those pixels as the “refining interval”.

For example, a multiresolution algorithm with fixed depth that matches the points of two 256 × 256 pixels images, say *x*_0_ and *y*_0_, may use three pairs of images having, thus, level 3 of sizes 128 × 128, 64 × 64 and 32 × 32; we denote these pairs of images (*x_ℓ_*, *y_ℓ_*), 1 ≤ *ℓ* ≤ 3 respectively. Note that usually *x_ℓ_*(*i*, *j*) = (*x*_*ℓ−*1_(2*i*, 2*j*) + *x*_*ℓ−*1_(2*i* + 1, 2*j*) + *x*_*ℓ−*1_(2*i*, 2*j* + 1) + *x*_*ℓ−*1_(2*i* + 1, 2*j* + 1))*/*4, for every 1 ≤ *ℓ* ≤ 3, but other operators are also possible as will be seen in Section 4. In this case the window size is *w* = 2. The same transformation is recursively applied to *y*_0_ in order to obtain *y*_1_, *y*_2_ and *y*_3_. We omit the dependence of the coordinates (*i*, *j*) on the level *ℓ* for the sake of simplicity.

The classical approach would attempt to match all the 32 × 32 pixels of the pair (*x*_3_, *y*_3_) to, then, proceed to their refinement. The refinement of pixel *x*_3_(*i*, *j*) consists of correlating the values *x*_2_(2*i*, 2*j*), *x*_2_(2*i* + 1, 2*j*), *x*_2_(2*i*, 2*j* + 1) and *x*_2_(2*i* + 1, 2*j* + 1) with the pixels within the refining interval around the matching point of *y*_2_. This is repeated until the matching is done on the (*x*_0_, *y*_0_) pair, obtaining the final result.

This approach is known to be faster than the brute force search on (*x*_0_, *y*_0_) (plain correlation). In fact, on the extreme case, where the images are squares and the smallest ones are single pixels, it requires *w*^2^ log(*w*) correlations, were *w* is the window size, thus its complexity is *O*(*w*^2^ log(*w*)). Of course, there is the time used for building the pyramid. So, to determine final algorithm complexity, one must add the complexity for building the pyramid, which is *O*(*w*^2^) + *O*(*w*^2^*/*4) + ⋯ + *O*(*w*^2^*/w*^2^), with the complexity of the matching, given above, which results anyway in *O*(*w*^2^ log(*w*)).

Reducing the search interval is not very efficient at improving this algorithm, since the gain in operations comes at the expense of more errors. Often, important characteristics are lost in the smaller images, reducing correlation precision. Those errors can sometimes be alleviated by a larger refining interval, which increases the execution time.

In practice, some implementations relate that the processing time used for building the multiresolution pyramid often compensates for the time gained on optimizing the correlations [22]. This basic multiresolution matching is seldom used in current applications [21].

## 4. Proposal: Multiresolution Matching with Variable Depth

As previously seen, plain correlation matching is very expensive and prone to generating errors such as ambiguity or lack of correspondence when there is not enough texture detail. On the other hand, multiresolution matching with fixed depth also tends to generate errors, but most of the pixels are still near correctly assigned. Also, the number of errors increases with the depth of the algorithm, since they are due to loss of information on the coarser images.

To get the best of both algorithms, one could assign for each pixel a different level: hard-to-compute positions should be treated at the highest resolution, while the others could be treated at an optimum, coarser level with just enough information. This adaptive approach, which is the proposed multiresolution matching with variable depth, will be shown to be able to reduce errors while still requiring less computational effort. The optimal level is computed on one of the images, and then each displacement is calculated in the same way as is done on the fixed depth algorithm.

An heuristic is, then, needed to calculate the desired depth. Also, we need to generate the small resolution images.

The proposed algorithm uses, for each image, a scale pyramid with several resolution versions of the original image, and one or more detail images. Scale images are obtained by a sub-band filter applied to the original images, while detail images are obtained by filtering the contents of the same level, scale image. We assessed two distinct approaches for the pyramid creation that differentiate mainly in the manner that the detail images are calculated: wavelets, and by Gaussian and Laplacian operators. They are described in the following sections.

### 4.1. Building the pyramids with wavelets

**Figure 1.**
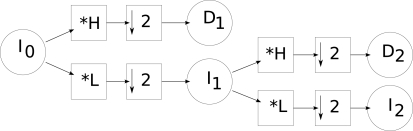
Creation of a pyramid with wavelet transform.

We used a discrete wavelet transform to build the pyramids. With this approach, in a given level *i*, the scale image of the pyramid (*I_i_*) is obtained by applying a low pass filter (*L*) to the scale image of level *i* − 1 followed by a decimation (↓). Detail images *D_i_* (with vertical, horizontal and diagonal details) are calculated using high-pass filters applied to the scale image of level *i* − 1 followed by a decimation. Figure 1 shows the schema for calculating a wavelet pyramid of level 2. We used the Daubechies and Haar bases [63].

### 4.2. Building the pyramids with Gaussian and Laplacian operators

We build two multiresolution pyramids by successively convolving the previous images with the low-pass Gaussian (ϒ*_G_*) and high-pass Laplacian masks (ϒ*_L_*) defined in Equations (2), and then decimating:

(2) $${ϒ}_{G}=\frac{1}{16}\left[\begin{array}{ccc} 1 & 2 & 1\\ 2 & 4 & 2\\ 1 & 2 & 1 \end{array}\right],\;\;\;\;{ϒ}_{L}=\left[\begin{array}{ccc} -1 & 1 & -1\\ 1 & 4 & 1\\ -1 & 1 & -1 \end{array}\right].$$

**Figure 2.**
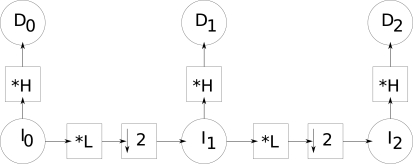
Illustration of the creation of a pyramid with three levels.

With this, we generate a pyramid of images and another of details. Figure 2 illustrates this filtering process used for the creation of a pyramid with three levels. By convolving the original image *I*_0_ with the high-pass filter (*H* mask), image *D*_0_ is generated. *I*_0_ is then convolved with the low-pass filter defined by the mask *L*, and decimated by a factor 2, which generates *I*_1_. This last image is then convolved again with the high-pass filter defined by the mask *H*, generating *D*_1_. A second low-pass filter (*L*) followed by a decimation, applied to *I*_1_, generates image *I*_2_, which is finally filtered by *H* generating *D*_2_.

These two pyramids are able to retain enough information in order to allow an efficient search for matching points.

The use of a sub-band filtering makes this algorithm much faster than the one proposed by Hoff and Ahuja [37], by removing the bottle-neck which is filtering. This fact, plus a lower error rate, allows to use a smaller refinement interval, which makes the multiresolution matching with variable depth much faster than the one with fixed depth and than the simple correlation approach in the original images.

Due to decimation, the construction of the scale images of the pyramid cannot be made shift-invariant. However, the detail images can be shift-invariant and this is a key difference between the two techniques. In the case of wavelets, the detail images are sensitive to shifts, but with 2D filtering they are invariant.

The wavelet transform is invertible. 2D filtering based transform is invertible only if both the high-pass and low-pass filters are ideal filters [64], which amounts to using convolution masks of the size of the original image. In order to be economic, small masks are employed and, therefore, this transformation is not invertible.

### 4.3. Desired level calculation

We use a propositional logic based on fuzzy evidence to derive a heuristic for calculating the desired level from which the matching will be performed. Such level is the coarsest one that can be labeled as “reliable”, in the sense that it provides enough information for the matching.

Fuzzy logic is composed of propositions *P* with continuous rather than binary truth values *μ*(*P*) ∈ [0, 1]. We used the following operators on those propositions: “¬”, where *μ*(¬*P*) = 1 − *μ*(*P*), “∧”, where *μ*(*A* ∧ *B*) = min(*μ*(*A*), *μ*(*B*)), “∨”, where *μ*(*A* ∨ *B*) = max(*μ*(*A*), *μ*(*B*)), “⇒”, where (*A* ⇒ *B*) ⇔ (*μ*(*B*) ≥ *μ*(*A*)) and “⇏”, where (*A* ⇏ *B*) ⇔ (*μ*(*A*) *> μ*(*B*)).

We define a predicate *σ_ℓ_*(*i*, *j*) meaning “the classification of the block at position (*i*, *j*) and level *ℓ* is not reliable”. This predicate must satisfy the following conditions:

- If the detail at (*i*, *j*) is zero, the classification is reliable: *D*(*i*, *j*) ≠ 0 ⇒ *σ_ℓ_*(*i*, *j*), where *D* is the amount of detail available.
- The deeper the classification the less reliable it is: if *K_ℓ_*_+1_(*i*, *j*) is the set of pixels at level *ℓ* + 1 that collapse into pixel (*i*, *j*) at level *ℓ*, we have that ∨_*v*∈*K*_*ℓ*+1_(*i,j*)_ *σ_ℓ_*(*v*) ⇒ *σ*_*ℓ*+1_(*i*, *j*).

Lack of texture details may cause accumulation of small errors, but this conflicts with getting always some minimum texture at the coarsest level, so we opted not to accumulate errors.

Because short execution time is our main objective, the heuristic has to be easy to compute by general purpose computers, leading to Equation (3):

(3) $$\sigma_{\ell }\;(i,j)=\left(\underset{(i,j)\in K}{\vee }\sigma_{\ell -1}\;(i,j)\right)\vee D(i,j)\neq 0.$$

We define, for any *a* ∈ [−1, 1], *μ*(*a* ≠ 0) = |*a*|, completely specifying the heuristic. Defining a dependability threshold *δ* ∈ [0, 1], our desired level for each pixel is the maximum level *ℓ* for which *δ* ⇒ *σ_ℓ_*.

**Figure 3.**
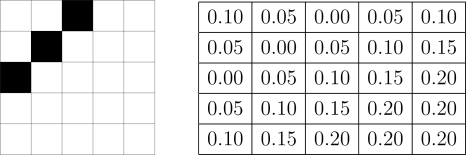
Cartoon image and *δ* map.

The ideal values of *δ* depend on the amount of detail in the image and, in principle, different values of *δ* should be associated to each pixel. For example, an image with substantial detail (texture) would be better treated at highest resolution, i.e., it should have values of *δ* very close to zero. Flat images with little detail could be dealt with at very coarse resolution without loosing information, *i.e.*, with *δ* close to 1. Figure 3 illustrates this with a 5 × 5 image, where each pixel has a different *δ* associated to it; notice that the smallest values are associated to the border, where there is detail that would be lost if treated at a coarse resolution.

However, the amount of texture is not known a priori. So, in this work, an empirically value is assigned for *δ* and kept constant for the whole image. In practice, we found that values greater than 0.2, cause the algorithm not to perform well, as it will be seen in the experiments.

### 4.4. Execution time considerations

The fuzzy heuristic presented above is able to assign a proper level to every pixel of an image, identifying detailed and flat areas. A successful technique for our purposes should be able to detect the level of detail of each image region based on texture. Flat regions should be treated at coarser, *i.e.*, higher levels of the pyramid (at the pyramid top) since they carry less information than detailed regions, which should be treated at lower levels (at the pyramid basis).

As it will be shown, at the coarsest level, the variable depth multiresolution matching also makes less mistakes than the fixed depth approaches. Because of that, we were able to obtain good results even with a refining interval as small as four pixels wide, leading to very fast execution.

The implementation of our proposal requires complex memory management that allocates and frees amounts of memory equivalent of several pages of the most common processors. Most operating systems lose performance on such conditions. So, also as a contribution of this work, we implemented a secondary memory management strategy that uses a buffer allocated only once at the beginning of execution. This pre-allocated memory is then managed by our procedure avoiding several calls to the operating system to perform this task. This approach alleviates the execution time, rendering a still faster procedure.

## 5. System Architecture

**Figure 4.**
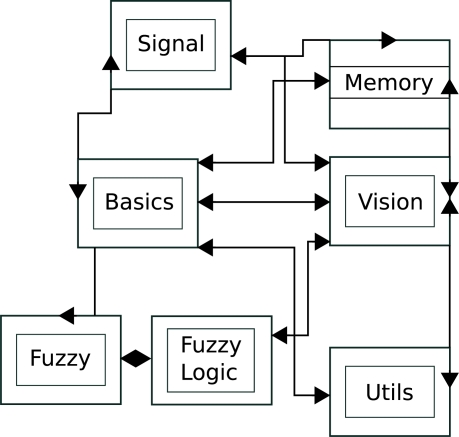
Scheme of the software architecture.

The proposed technique was implemented as a C++ library and a collection of test programs. This library generates disparity maps using the default correlation method and our approach, using multi-resolution with variable depth, considering or not a search interval. Due to the complexity of this library, its implementation was divided in several modules as shown in Figure 4.

The *Basics* module contains common classes used by other modules. *Signal* is composed by classes that store and operate on images. *Memory* comprises the classes responsible for memory management and for the implementation of the data structures used. *FuzzyLogic* implements the fuzzy decision given in Equation (3), and disparity calculation. *Vision* is composed by classes that implement the stereo vision algorithms and related functions. *Utils* packs auxiliary code used for the manipulation of the test images and extraction of results from data.

Each module is detailed in the following.

### 5.1. Module Basics

This module contains the library ops.h, that implements operations which are required in almost every stage. It also has the classes Position, that stores a position of type “(row, column)”, Window, that defines a rectangular area of interest, and Interval, that defines a connected subset of integer values. Classes Window and Interval also store some pre-calculated values used to accelerate the matching.

### 5.2. Module Signal

This module contains the template Image, and classes that specialize Pixel: ColorPixel, BWPixel, PositionPixel, BWLabel and ColorLabel. Image<PixType> has an array of elements of type PixType that represents the pixels. This template implements operations for image reading and writing images in PGM and PPM formats, and also guarantees access to operations in pixels and the wavelet transform.

Pixel provides arithmetic operators used in transformations and convolutions, besides methods for extracting data. Types ColorPixel and BWPixel implement pixels for color and monochromatic images. Types ColorLabel and BWLabel implement color and monochromatic pixels also, but with an integer identification code (id). PositionPixel implements a gray level pixel with integer value; it stores the final disparity map values and an integer id.

The data structures that store pyramids of images, regardless the technique (wavelets or 2D filtering), are created by the classes ImgPair and LowHigh. The former returns the first pair of images in the pyramid, while the latter builds the remaining pairs. Classes ImgSet and ImgListSet implement the data structure that contains the four images generated by wavelets transform and the lists of the images generated in a sequence of transformations, respectively.

Class DWT has values and methods used by the Daubechies wavelet transform. An object of class DWT has filters of a transformation implemented in another class; this strategy is adopted to avoid the use of a virtual class. Classes Haar and Daub4 implement the two types of wavelets used in this work, namely Daubechies and Haar.

### 5.3. Memory Module

The result of the heuristic that calculates the desired depth for each pixel requires a complex data structure. We implemented linked lists that contain objects of class Position. These lists have different formats in each execution of the matching requiring, thus, dynamical allocation of memory. A problem is that a list may use a large region of memory that may, sometimes, grow up to several megabytes. This is beyond the size of the memory page of most modern computer architectures, which is usually 16 Kb. As current operational systems usually lose performance as they allocate and free, repeatedly, such amounts of memory, we developed a memory managing system for our library. To do that, we created the class MemoryBuffer containing a buffer, which is allocated at the initialization, and resources for managing it.

By using the class MemoryBuffer, tailored to the needs of our library, program execution is much faster than by using the memory management provided by the operating system. The directive FAST_MEMORY, available at compiling time, makes memory management still faster by disabling the checking of buffer limit. When used through this library, all data stored in these buffers are calculated locally and not brought from other programs. We remark that this strategy presents low risk for the system security.

The class List implements a low-level list that can deal with allocated memory, with or without the aid of an object of the type MemoryBuffer. The other classes of this module are LinkedList and Stack, that implement high-level data structures (linked list and stack, respectively), useful for other modules of the library.

### 5.4. FuzzyLogic Module

Class Fuzzy represents the *fuzzy* hypotheses, with the following operators: ¬ (!), ⊕ (+), . (*), ∨ (|), ∧ (&), ⇒ (<), and ⇏ (>).

Class FuzzyImax also composes this module. It is responsible for calculating the desired depth for each pixel. The return value of this method is of type LinkedList<LinkedList<Position>>, where Position stores a position in image. The output is a list of depth levels. For each depth, there is a list of pixels where disparity calculations start from that depth.

Note that each image pixel can be represented in more than a depth. In such case, matching must be performed at the least resolution depth in which the pixel is found. For example, if the sixth element of the returned list has position (1, 1), this means that for all pixels in the original image that lie in positions (*x*, *y*), *x*, *y <* 2^6^, the greater level that can be used is 5 (starting from zero). It is possible for a pixel to appear twice in the list, for instance if position (2, 3) appears at the fourth list, for all pixels of the interval (x,y), 2 × 2^4^ ≤ *x <*3 ×2^4^, 3 × 2^4^ ≤ *y <*4 × 2^4^, that is, in the interval (*x*, *y*), *x*, *y <* 2^6^, the depth must be up to 3, and not 5 anymore.

The easiest way of obtaining depth for each level is, thus, by traveling this list starting from the less coarse level and marking positions already visited. For that, pixels of the type ColorLabel and BWLabel are used.

### 5.5. Using the library

The main classes for our application are LeftImax and PlainCorr, both derived from Vision. These classes implement the multiresolution with variable depth matching and the simple correlation methods. Objects of both classes are created using as parameters the left and right images, and the resulting image were disparity will be stored. Images can be created through allocation of a memory area or using an already allocated area. Image data are stored linewise as one-dimensional arrays.

Objects of classes LeftImax and PlainCorr can then be initialized with setWindow. For simple correlation, arguments are setWindow (Window C, Interval B), where C is the comparison window and B is the search interval. In this implementation, arguments are setWindow (Window C, Interval B, Interval R), where C and B are the same and R is the refining interval.

Classes Window and Interval define windows and intervals, respectively, as integer numbers. Windows can be created at any position, using Window (int rmin, int rmax, int cmin, int cmax), where rmin and rmax are the extreme lines that the window contains, and cmin and cmax the extreme columns. Intervals can be created in arbitrary positions; Interval (int min, int max) creates the interval [ min; max].

After windows are initialized, the matching is performed using match of LeftImax or PlainCorr. For plain correlation, this method does not receive arguments, and in multiresolution matching with variable depth it receives match ( Fuzzy *δ*) as argument, where *δ* is as defined in Equation (3). After matching is performed, disparities can be read at the resulting image.

Memory allocation is always done in a transparent way to the programmer. All necessary memory is allocated at the creation of the objects of classes LeftImax and PlainCorr. Garbage collection, however, is not supported. This is not a problem in most applications, but might be an issue when dealing with images from several pairs of different cameras. The constructor of class Fuzzy receives only an argument of type double that represents, in this case, *μ*(*δ*).

## 6. Experimental Results

**Figure 5.**
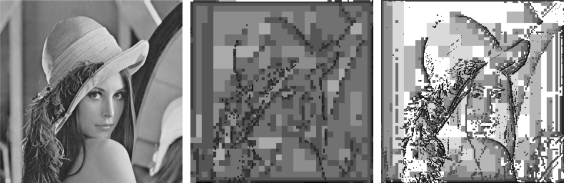
Computed pyramids. Left to right: original image, Daubechies wavelet levels, and levels computed by our proposal.

An example of pyramids is shown in Figure 5. The image to the left is the well known Lena data set, used as a benchmark in many applications because it presents both flat and detailed areas. Middle and right of Figure 5 show the levels computed by the Daubechies wavelet decomposition (of size 4) and by our approach (computed using *μ*(*δ*) = 0.2), respectively; darker pixels are coarser and, thus, require more time to process.

We performed stereo measures using both approaches, but the use of wavelets (both Daubechies and Haar) for computing the pyramid turns out not being as efficient to subsequent phases as our proposal. Differently from other works [31, 65], our approach employs the detail coefficients being, thus, more vulnerable to problems due to the transformation not being shift invariant. So we adopt the approach that uses the high and low pass filtered pyramid due to its better performance.

**Figure 6.**
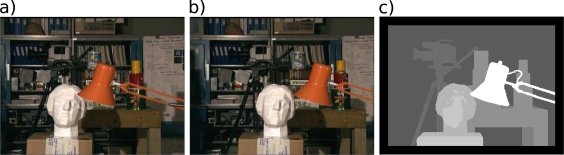
Tsukuba data set. From left to right: left image, right image, desired disparity map.

**Figure 7.**
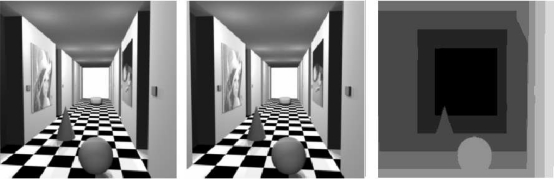
Tsukuba data set. From left to right: left image, right image, desired disparity map.

We contrasted plain correlation and multiresolution with variable depth matching using them on two well known pair of images, namely the Tsukuba and Corridor data sets, and comparing the results with the available ground truth. Figures 6 and 7 show the pairs, along with the desired disparity maps (ground truths).

The matching results are compared with the desired ones in two ways, by visual analysis and by using an error metric. We use the mean error (Equation (4)) and its standard deviation (Equation (5)) as measures of precision:

(4) $$d=\frac{\sum_{i,j}\;(O(i,j)-D(i,j))}{N},$$

(5) $$s=\frac{1}{N}\sqrt{\sum_{i,j}{\left(O(i,j)-D(i,j)\right)}^{2}.,}$$

where *O* and *D* denote, respectively, the observed and desired disparity maps.

These error measurements are insensitive to the shape of the objects but are not so good for describing the quality of results on regions close to borders and edges. In this case, we use visual inspection that is, on the other hand, good in these tasks at the expense of being subjective. We therefore use these two complementary methods.

We used square correlation windows of side 3, 5, 7, 9, and 11 pixels, in order to test our approach with more than one window size. This means that, for a certain resolution level, given a pixel in one image (say the left) to be matched to a pixel in the other image (say right), a template window of a specified size will be taken around the pixel in the left image. Correlation measures will be calculated for this window with several windows of the same size taken around pixels in the epipolar line in the right image, within a certain search interval. When using the plain correlation algorithm, if a search interval is defined, it is always 70 pixels wide (not the whole epipolar line). We remark that, even with this optimization, plain correlation is still a time consuming algorithm. On the multiresolution matching, the refining interval is always 4 pixels wide.

### 6.1. Comparing Multiresolution Algorithms

We performed tests with two versions of our multiresolution matching. The first uses only scale images in all levels based on correlation measures. The second uses the detail images in each level and the scale images at the coarsest level, since at this level there is less detail.

**Figure 8.**
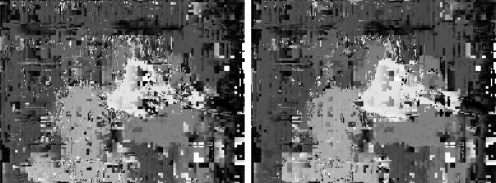
Disparity maps generated by multiresolution matching using the detail images at the coarsest level (level), and using always the scale images (right).

**Figure 9.**
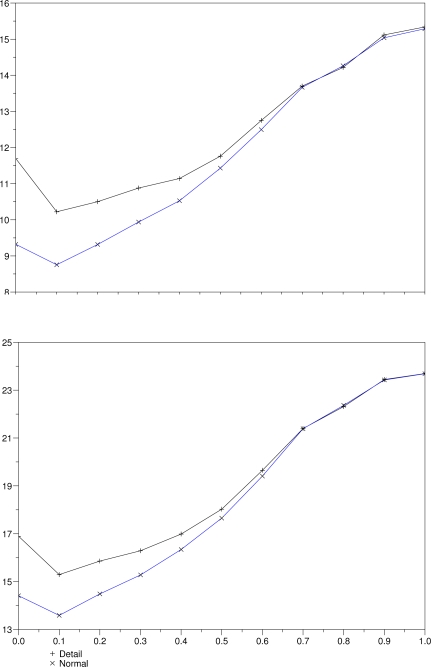
Errors measured with both algorithms: mean distance *d* (left) and standard deviation *s* (right).

Disparity maps generated by both versions of our multiresolution algorithm are shown in Figure 8. These results are obtained with a correlation window of size 3 and a threshold *δ* = 0.3. Note that borders and edges obtained by the algorithm that uses detail coefficients are sharper and better defined than the ones produced by the other technique, which only uses scale images. Besides that, the overall aspect of the former disparity map is better than the latter. Figure 9 shows average measures of the errors obtained with several thresholds for both versions, keeping the correlation window at size 3. The minimum in both lines near the origin indicates that the threshold *δ* = 0.3 produced less errors. The use of scale images at all levels produces results with less errors, what is represented by the bottom lines in both graphs.

With the new fuzzy heuristic, multi-resolution matching is likely to start at the lowest level where there is a border adjacent to the pixel under assessment. The correlation of the images at the coarsest depth is, thus, highly prone to errors due to occlusions. Matching the details, instead of the raw images, should, in principle, lead to higher resistance to occlusions. That behavior was confirmed in our experiments, as the results obtained matching the scale images at each level were consistently better than those that employed detail information.

### 6.2. Comparing Multiresolution and Plain Correlation

**Figure 10.**
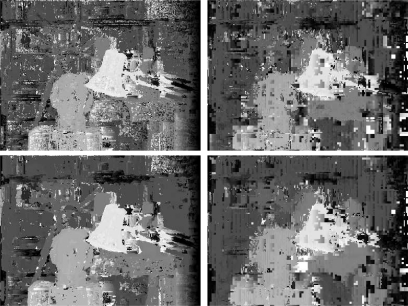
Disparities obtained by plain correlation (right) and multiresolution (left) with correlation windows of size 3 (top) and 5 (bottom) pixels, using *δ* = 0.3.

Here we contrast plain correlation with multiresolution algorithm. Disparity maps obtained by both algorithms are shown in Figure 10.

**Figure 11.**
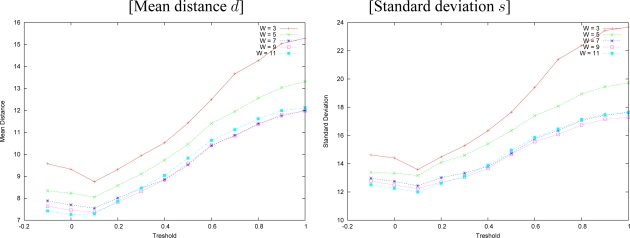
Measured errors for multiresolution with variable depth: Tsukuba pair.

**Figure 12.**
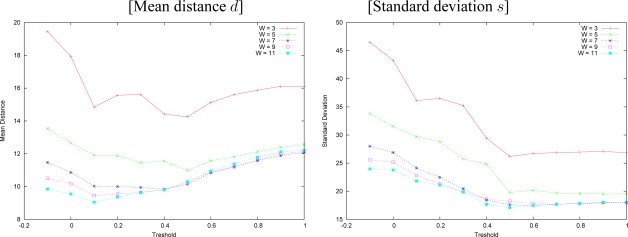
Measured errors for plain correlation with no search interval: Tsukuba pair.

We made experiments with both approaches for window sizes of 3, 5, 7, 9 and 11. Standard deviation and mean distance of the measured errors for multiresolution approach with variable depth are shown in Figure 11. The same error measures produced by the technique without search interval are shown in Figure 12 for the same window sizes.

We observe that larger windows generate smaller errors in both approaches. Multiresolution incurred in smaller errors than plain correlation in most cases, and it made mistakes as often as the plain correlation. Plain correlation produces errors distributed on bigger areas than our algorithm, which is hard to visualize in the disparity figures. By the results, on the overall, our approach performed better than plain correlation.

**Figure 13.**
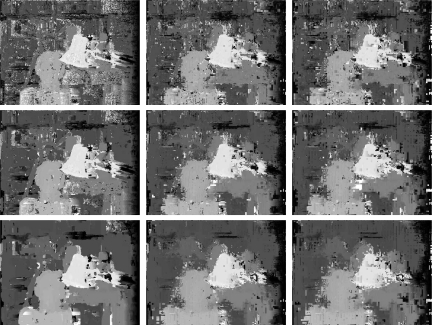
Visual comparison between disparity maps generated by correlation (right column) and multiresolution matching with *δ* ∈ {0.1, 0.2} (middle and left columns, respectively), Tsukuba data set, using windows of size 3, 5, 9 (top, middle and bottom rows, resp.), 4 pixels search interval.

**Figure 14.**
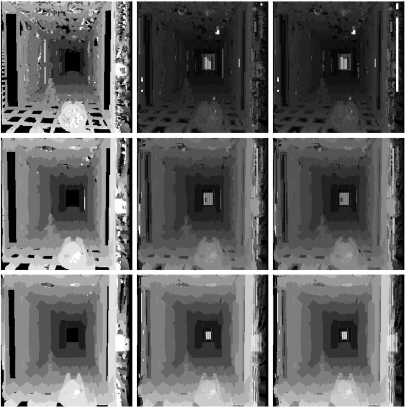
Visual comparison for the Corridor images between disparity maps generated by correlation (right column) and multiresolution matching with *δ* ∈ {0.1, 0.2} (middle and left columns, respectively), using windows of size 5, 9, 13 (top, middle and bottom rows, resp.), 10 pixels search interval

Figure 13 shows a comparison between the matching using the two algorithms (plain correlation and ours, with threshold *δ* = 0.1, 0.2) for the Tsukuba images, while Figure 14 shows the same comparison applied to the Corridor images.

**Figure 15.**
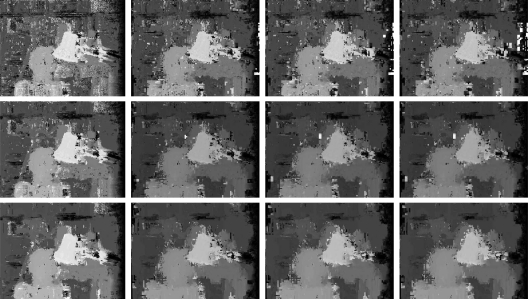
Disparity maps generated by multiresolution matching with *δ* ∈ {0, 0.2, 0.3, 0.4} (columns from left to right) and windows of size 3, 5, 7 (rows from top to bottom), 6 pixels search interval.

Figure 15 shows results of varying *δ*, with a search interval of 6 pixels wide.

**Figure 16.**
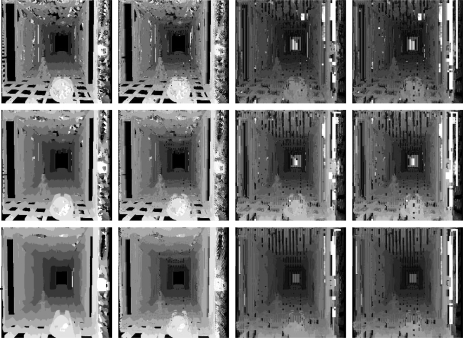
Disparity maps generated, Corridor, by generated by correlation (right column) and multiresolution matching multiresolution matching with *δ* ∈ {0, 0.1, 0.2} (columns from left to right), windows of size 5, 7, 11 (from top to bottom), refinement windows of 4 pixels.

**Figure 17.**
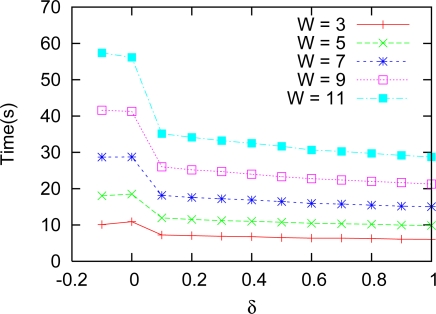
Time needed for computing the disparity by our approach in the Corridor pair.

**Figure 18.**
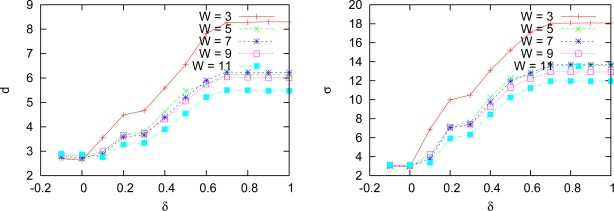
Error and standard variation for the Corridor images.

We tested both algorithms also in the Corridor image, and the results are shown in Figure 16. In this case, a search interval of 10 pixels was imposed, a refinement interval of 4 and 6 pixels and square search window sizes of 5, 7, and 11 pixels. We tried with several limits (*δ*). Figure 17 shows the time necessary for running this experiment. The best result of the matching is achieved for *δ* = 0.05 and the best times start at *δ* = 0.1. So, one has to weight between precision and time. The result of the matching is still better than plain correlation for *δ* = 0.05, whose error and standard deviation are shown in Figure 18.

**Figure 19.**
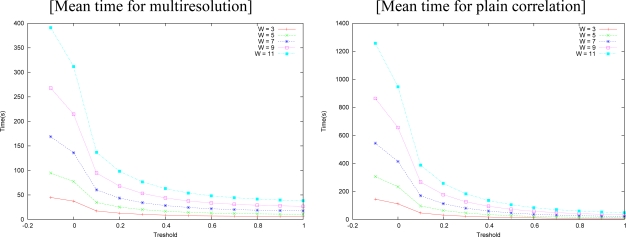
Required time.

The time needed for the matching processes is shown in Figure 19 as a function of the threshold (*δ*). Multiresolution matching was consistently faster than plain correlation. It should be remarked that the execution time of our algorithm is much shorter than the plain correlation, on all thresholds, and it is even faster at small thresholds. Note that smaller correlation windows need less time. One has to weight between precision and available time when deciding the size to be used. Plain correlation errors usually increase a little from *δ* = 0, but they fall at near the same or smaller values near *δ* = 0.3, which seems to be an optimum threshold.

## 7. Discussion and Conclusions

We have proposed a new approach to stereo matching using multiresolution in which the level with which to start is variable as a function of the images content. That is, in a given region, for example a smooth one without edges, our algorithm starts in coarser (deeper) levels in order to improve precision; in regions with edges or well textured, it starts in finer (lower) levels reaching, thus, better execution time. Our approach is based on fuzzy logic, in order to define the level with which to start the matching, for each image region. By the results, this fuzzy logic decision process has proven to be excellent for this calculation.

The ideal value for *δ* depends on the image content and on lighting conditions. Such value should, in principle, be tuned automatically or dynamically, as a function of the amount of texture, both locally and globally. Such measure can be performed by means of using the operators described in [66, 67], or by calculating the image focus [68, 69]. Our best results were obtained in the vicinity of *δ* = 0.1, and they are robust in the interval [0.05, 0.3).

The ideal window size is also dependent on the amount of texture in the original image pair. This parameter and can also be estimated using a similar procedure as the one proposed for *δ* [70].

Initial experiments using wavelets in order to calculate the multiresolution pyramid were not good enough due to the use of the detail coefficients. We then decided to apply a sub-band filtering based on a low pass Gaussian and a high pass Laplacian masks to generate the two multiresolution pyramids: one of images and other of details. With this approach, stereo matching performed much better, that is, faster and with better precision in stereo measurements.

The main contribution of this work is the multiresolution approach, which differs from usual methods, as seen above, by using a new fuzzy logic heuristic for calculating the starting level.

Our algorithm was able to generate disparity maps faster than plain correlation, with smaller errors. We conjecture that the use of Gaussian and Laplacian masks reduced even further the errors that occur close to borders. That is, those filters have a smoothing effect in such regions, allowing the algorithm to better treat occlusions.

Recent research on stereo matching based on multi-resolution and fuzzy techniques has been conducted, as discussed in Section 2. However, when facing the problem of real-time stereo matching, as in robotics vision, correlation based algorithms are known to be the best [71]. Despite that, in order to validate our approach with respect to techniques other than plain correlation, we tested two procedures, namely, a very fast multi-resolution approach [17], and one based on fuzzy logic [41].

In the fast multi-resolution approach [17], we used 4 levels with images of sizes 96 × 72 and 64 × 48 pixels. Average errors of 30 and 35 pixels were observed, with standard deviation of 65 and 54, respectively. The time spent for disparity calculation was 5 and 12 milliseconds, making the technique a very efficient algorithm that runs in real time. Despite its efficiency, it has poor precision.

**Figure 20.**
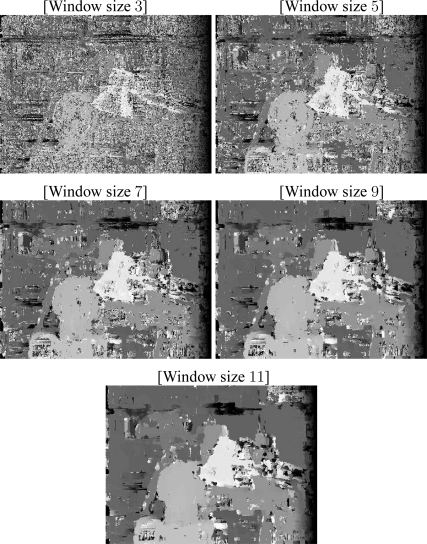
Disparity maps, Kumar and Chatterji algorithm, for window of sizes 3, 5, 7, 9, and 11 (from top to bottom).

The fuzzy approach by Kumar and Chatterji [41] leads to errors and time execution also bigger than the ones produced by our approach. We tested with a search interval of 64 pixels wide, with windows of sizes 3, 5, 7, 9 and 11, as reported in Table 1. This method produces a mean error of 14 pixels with standard deviation 19, and time execution of 21 seconds when using window size of 3 × 3. When using a window of size 11 × 11, the error decreases to 7 with standard deviation 12, however the time execution increases to 241 seconds. Figure 20 shows the disparity maps obtained with this approach (from top to bottom, window sizes of 3, 5, 7, 9 and 11 are shown).

These two techniques are, therefore, outperformed by our proposal when both precision and performance are required.

## Figures and Tables

**Table 1.** Performance measures, Kumar and Chatterji’s algorithm, as a function of the window size.

Window	Mean	Standard	Execution

Size	Error	Deviation	Time
3	14.12	19.74	21.00
5	10.66	16.04	53.31
7	8.92	13.96	98.48
9	8.09	13.07	161.06
11	7.61	12.56	241.15

## References

[1] Horn B.K.P. (1986). Robot Vision.

[2] Marr D. (1982). Vision — A Computational Investigation into the Human Representation and Processing of Visual Information.

[3] Lotti J.L., Giraudon G., Desachy J. (1994). Correlation algorithm with adaptive window for aerial image in stereo vision. Image and Signal Processing for Remote Sensing.

[4] Hespanha J., Dods Z., Hagger G., Morse A. Decidability of robot positioning tasks using stereo vision system.

[5] Hubber E., Kortenkamp D. Using stereo vision to pursue moving agents with a mobile robot.

[6] Matsumoto Y., Shibata T., Sakai K., Inaba M., Inoue H. Real-time color stereo vision system for a mobile robot based on field multiplexing.

[7] Murray D., Little J. (2000). Using real-time stereo vision for mobile robot navigation. Auton. Rob.

[8] Ballard D.H., Brown C.M. (1982). Computer Vision.

[9] Marr D., Poggio T. (1976). Cooperative computation of stereo disparity. Science.

[10] Marr D., Poggio T. (1979). A computational theory of human stereo vision. Proc. Royal Soc. London.

[11] Trucco E., Verri A. (1998). Introductory Techniques for 3D Computer Vision.

[12] Fleet D.J., Wagner H., Heeger D.J. (1997). Neural encoding of binocular disparity: Energy models, position shifts and phase shifts. Vis. Res.

[13] Gonçalves L.M.G., Oliveira A.A.F. Pipeline stereo matching in binary images.

[14] Oliveira A.A.F., Gonçalves L.M., Matias I.O. Enhancing the Volumetric Approach to Stereo Matching.

[15] Theimer W.M., Mallot H.A. (1994). Phase-based binocular vergence control and depth reconstruction using active vision. Comput. Vis. Graph., Image Process.: Image Underst.

[16] Zitnick C.L., Kanade T. (2000). A cooperative algorithm for stereo matching and occlusion detection. Trans. Pattern Anal. Mach. Intell.

[17] Gonçalves L.M.G., Oliveira A.A., Grupen R.A., Wheeler D., Fagg A. (2000). Tracing patterns and attention: humanoid robot cognition. IEEE Intell. Syst. Their Appl.

[18] Nishihara K. (1984). Practical Real-Time Stereo Matcher.

[19] Ohta Y., Kanade T. (1985). Stereo by Intra and inter-scanline searching using dynamic programming. Trans. Pattern Anal. Mach. Intell.

[20] Ullman S. (1996). High-level Vision: Object Recognition and Visual Cognition.

[21] Segundo S.S., Bezerra J.P., Silveira R.W., Gonçalves L.M.G. Development of a multiresolution stereo vision system in real time.

[22] Hirschmüller H. Improvements in real-time correlation-based stereo vision.

[23] Sun C. (2002). Fast stereo matching using rectangular subregioning and 3d maximum-surface techniques. Int. J. Comput. Vis.

[24] Witkin A.P. Scale-space filtering.

[25] Lindeberg T. (1994). Scale-Space Theory in Computer Vision.

[26] Burt P., Adelson T. (1983). The Laplacian pyramid as a compact image code. IEEE Trans. Commun.

[27] Uhr L. (1972). Layered ‘recognition cone’ networks that preprocess, classify and describe. IEEE Trans. Comput.

[28] Itti L., Koch C., Niebur E. (1998). A model of saliency-based visual attention for rapid scene analysis. IEEE Trans. Patten Anal. Mach. Intell.

[29] Sandon P.A. Logarithmic search in a winner-take-all network.

[30] Daubechies I. (1988). Orthonormal bases of compactly supported wavelets. Commun. Pure Appl. Math.

[31] Mallat S. (1996). Wavelets for a vision. Proc. IEEE.

[32] Iocchi L., Konolige K. A multiresolution stereo vision for mobile robots.

[33] Magarey J., Dick A. Multiresolution stereo image matching using complex wavelets.

[34] Pan H.P. (1996). General stereo image matching using symetric complex wavelets. Wavelets Aplications in Signal and Image Processing.

[35] Gonçalves L.M.G., Grupen R.A. (2000). Towards a real-time framework for visual monitoring tasks.

[36] Piater J., Ramamritham K., Grupen R.A. Learning real-time stereo vergence control.

[37] Hoff W., Ahuja N. (1989). Surfaces from stereo: integrating feature maching, disparity estimation, and contour detection. IEEE Trans. Pattern Anal. Mach. Intell.

[38] Udupa J.K., Grevera G.J. (2002). Go digital, go fuzzy. Pattern Recogn. Lett.

[39] Bigand A., Bouwmans T., Dubus J.P. (2001). A new stereomatching algorithm based on linear features and the fuzzy integral. Pattern Recogn. Lett.

[40] Kim D.H., Choi W.Y., Park R.H. (1992). Stereo matching technique based on the theory of possibility. Pattern Recogn. Lett.

[41] Kumar S.S., Chatterji B.N. (2002). Stereo matching algorithms based on fuzzy approach. Int. J. Pattern Recogn. Artif. Intell.

[42] Pajares G., de la Cruz J.M. (2000). A new learning strategy for stereo matching derived from a fuzzy clustering method. Fuzzy Sets Syst.

[43] Pajares G., de la Cruz J.M. (2006). Fuzzy cognitive maps for stereovision matching. Pattern Recogn.

[44] Sainarayanan G., Nagarajan R., Yaacob S. (2007). Fuzzy image processing scheme for autonomous navigation of human blind. Appl. Soft Comput.

[45] Shamir L. (2007). A proposed stereo matching algorithm for noisy sets of color images. Comput. Geosc.

[46] Tolt G., Kalaykov I. (2006). Measures based on fuzzy similarity for stereo matching of color images. Soft Comput.

[47] Doulamis N.D., Doulamis A.D., Avrithis Y.S., Ntalianis K.S., Kollias S.D. (2000). Efficient summarization of stereoscopic video sequences. IEEE Trans. Circ. Syst. Video Technol.

[48] McCane B., Caelli T., DeVel O. (1997). Learning to recognize 3D objects using sparse depth and intensity information. Int. J. Pattern Recogn. Artif. Intell.

[49] Nagata T., Zha H.B. (1991). Recognizing and locating a known object from multiple images. IEEE Trans. Rob. Autom.

[50] Nagarajan R., Sainarayanan G., Yaacob S., Porle R.R. (2005). Fuzzy-rule-based object identification methodology for NAVI system. EURASIP Journal on Applied Signal Processing.

[51] Chatterji G.J., Chatterji B.N. (2004). Fuzzy compactness based adaptive window approach for image matching in stereo vision. Neural Inf. Process.

[52] Aja-Fernandez S., Alberola-Lopez C., Ruiz-Alzola J. (2003). A fuzzy-controlled Kalman filter applied to stereo-visual tracking schemes. Signal Process.

[53] Chow Y.H., Chung R. (2002). VisionBug: A hexapod robot controlled by stereo cameras. Auton. Rob.

[54] Marichal G.N., Toledo J., Acosta L., Gonzalez E.J., Coll G. (2007). A neuro-fuzzy method applied to the motors of a stereovision system. Eng. Appl. Artif. Intell.

[55] Medeiros M.D., Gonçalves L.M. A fuzzy approach to stereo vision using pyramidal images with different starting level.

[56] Tsotos J.K. A complexity level analysis of vision.

[57] Tsotsos J.K. (1985). Knowledge organization and its role in representation and interpretation for time-varying data: the ALVEN system. Comput. Intell.

[58] Burt P. (1988). Smart sensing within a pyramid vision machine. Proc. IEEE.

[59] Sandon P. (1990). Simulating visual attention. J. Cogn. Neurosci.

[60] Tsotsos J., Culhane S., Wai W., Lai Y., Davis N., Nuflo F. (1995). Modeling visual attention via selective tuning. Artif. Intell. Mag.

[61] Lindeberg T. (1998). Feature detection with automatic scale selection. Int. J. Comput. Vis.

[62] Lowe D.G. (2004). Distinctive image features from scale-invariant keypoints. Int. J. Comput. Vis.

[63] Mallat S. (1999). A Wavelet Tour Of Signal Processing.

[64] Gonzalez R.C., Woods R.E. (1992). Digital Image Processing.

[65] Moon P., de Jager G. An investigation into the applicability of the wavelet transform to digital stereo matching.

[66] Karu K., Jain A.K., Bolles R.M. (1996). Is there any texture in the image?. Pattern Recogn.

[67] Malik J., Belongie S., Leung T., Shi J. (2001). Contour and texture analysis for image segmentation. Int. J. Comput. Vis.

[68] Ahmad M.B. Focus measure operator using 3D gradient.

[69] Favaro P., Soatto S. (2005). A geometric approach to shape from defocus. IEEE Trans. Pattern Anal. Mach. Intell.

[70] Woo D.M., Schultz H., Riseman E., Hanson A. (2005). Performance of correlation-based stereo algorithm with respect to the change of the window size.

[71] Yoon S., Park S.K., Kang S., Kwak Y.K. (2005). Fast correlation-based stereo matching with the reduction of systematic errors. Pattern Recogn. Lett.

